# Clinicopathological characteristics and outcomes of eosinophilic cystitis: A retrospective study

**DOI:** 10.1016/j.amsu.2021.102626

**Published:** 2021-07-28

**Authors:** Abdulkarim Hasan, Ibrahim Abdel-Al, Khalid Nafie, Mahmoud F. Rashad, Hesham Abozied, Mohammed E.A. Elhussiny, Ahmed Rabie, Ali A. Rabaan, Manar K. Abd Elnabi, Mohammed S. Abdelwahed, Mohammed A. Ahmed, Yasien Mohammed

**Affiliations:** a Department of Pathology, Faculty of Medicine, Al-Azhar University, Cairo, Egypt; b Laboratory & Blood Bank Department, Prince Mishari Bin Saud Hospital, Baljurashi, Saudi Arabia; c Department of Urology, Faculty of Medicine, Al-Azhar University, Assiut Branch, Egypt; d Department of Urology, Faculty of Medicine, Al-Azhar University, Cairo, Egypt; e Department of Histology, Faculty of Medicine, Al-Azhar University, Cairo, Egypt; f Department of Pathology, Faculty of Medicine, Al-Azhar University, Damietta, Egypt; g Molecular Diagnostic Laboratory, Johns Hopkins Aramco Healthcare, Dhahran, Saudi Arabia; h Biofuels Institute, School of the Environment and Safety Engineering, Jiangsu University, Zhenjiang, China; i Botany Department, Faculty of Science, Tanta University, Tanta, Egypt; j Department of Pathology, Faculty of Medicine, University of Jeddah, Jeddah, Saudi Arabia; k Department of Pathology, Faculty of Medicine, Al-Azhar University, Assiut Branch, Egypt

**Keywords:** Eosinophilic cystitis, Bladder mass, Histopathology, Allergy

## Abstract

**Background:**

Eosinophilic cystitis (EC) is a rare inflammatory urinary bladder disorder whose etiology, pathogenesis, and treatment are unknown. The work aims to evaluate the clinical manifestations, cystoscopic characteristics, pathological features, treatment, and clinical outcome of EC patients.

**Materials and methods:**

The clinical records and histopathology material of 22 patients diagnosed as EC during ten years were reviewed and analyzed for patient's age, sex, clinical data, cystoscopic features, biopsy procedures, treatment plan, follow-up, and prognosis. Frequencies, normality tests, descriptive statistics, and correlations were run.

**Results:**

The mean age of patients was 46.5 + 17 years, 12 females and 10 males. Regarding the patient's complaints, dysuria was the most frequent main symptom, followed by hematuria. On cystoscopic examination, bladder mass was seen in 54.5% of patients. Six patients (27.3%) were associated with different allergic diseases; however peripheral eosinophilia was shown in two patients (9.1%). All cases revealed predominance of eosinophilic infiltration on microscopic examination. The most commonly used medications were corticosteroids for 72.7% of patients with tapering dose giving a significant improvement with a recorded recurrence in one patient after 12 months from the first lesion.

**Conclusions:**

No specific clinical presentation for EC patients and histopathology is the standard diagnostic tool. Medical treatment including corticosteroids was the first line with good prognosis, although recurrence remains a possibility which emphasizes the importance of patients’ follow-up.

## Highlights

• Eosinophilic cystitis is a rare inflammatory disorder of the bladder.
• Etiology, pathogenesis, and treatment are unknown.
• A total of 22 patients were retrospectively studied and analyzed.
• Histopathological examination is standard tool for diagnosis.
• Oral steroids gave a significant improvement.

## 1 Introduction

Eosinophilic cystitis (EC) is a rare inflammatory disorder of the bladder whose etiology, pathogenesis, and treatment are unknown. The reported frequent symptoms of this disease include frequency, dysuria, and hematuria [1]. Although the etiology of EC is unclear, allergies or injuries of the bladder wall appear to be present in most patients [2]. The associated immunological factors of EC have been postulated to be immunoglobulin E (IgE) mediated formation of the antigen-antibody complexes that attract eosinophils to the bladder wall [3].

There are no specific imaging suggestive of EC, however, cystoscopy shows an inflamed, edematous ulcerated or necrotic mucosa, and sometimes polypoid areas or real tumor like mass may exist [4,5]. The histopathologic diagnosis is the main diagnostic tool for eosinophilic diseases including EC, in which, a transmural inflammation of the urinary bladder with predominant eosinophils is seen [[6], [7], [8]]. No treatment consensus exists due to its rare occurrence, but usually, the effective treatments include antihistamines and steroids [6]. In this study, we assess the clinicopathological characteristics and the clinical outcomes of EC in three referral hospitals during ten years.

## 2 Methods

This is a clinicopathological retrospective study performed at Al-Azhar hospitals (Cairo, Damietta, Assiut) and PMSH. After bioethical approval, the data of patients (n = 22) diagnosed with Eosinophilic cystitis (between 2011 and 2020) were obtained from the medical records and the pathology database. Patient's age, sex, symptoms, allergic history, clinical diagnosis, cystoscopic findings, relevant laboratory investigation results, treatment plan, and the clinical outcome during at least 6 months follow-up period were recorded. Hematoxylin and Eosin (H&E) ordinary stained slides were reviewed by three histopathologists. Exclusion criteria include patients with incomplete data, patients who did not complete 6 months follow-up duration, no available histopathology slides or paraffin blocks and lack of obtained confirmation of the final diagnosis by at least 2 pathologists (five cases were excluded).

All the inflammatory bladder biopsies in which more than 90% of the inflammatory infiltrate in the lamina propria and muscle comprised of eosinophils were confirmed as eosinophilic cystitis.

Statistics: Data was entered into the Excel program (Microsoft Corporation, Redmond, USA). Results were presented as the mean ± SD for age, frequencies and percentages were computed for the descriptive variables. A P-value of less than (0.05) was considered statistically significant. The results of this study come in accordance with STROCCS reporting statements [9].

## 3 Results

**Fig. 1:**
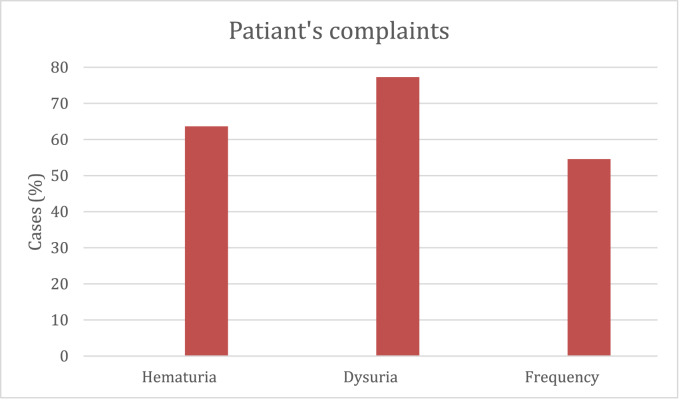
A bar chart showing the percent of the main patients' complaints.

The results in this study revealed an average age of 46.5 + 17 years ranged from 22 to 78 years, divided between 12 females and 10 males. Regarding the patient's complaints, dysuria was the most frequent main symptom, presented in 17 (77.2%) patients, followed by hematuria which was presented in 14 (63.6%) and frequency was presented in 12 (54.5%) patients (Fig. 1). On cystoscopic examination, bladder mass was the most common clinical finding as it was seen in 12 (54.5%) patients; 7 cases with solid bladder mass with a size from 1.3 to 2.5 cm with elevated hyperemic mucosa around the lesion (5 at dome and anterior wall and 2 cases at the posterior wall near to the bladder neck), most of them in (who developed with retention of urine), 5 cases post fixation of double j stent, was multiple papillary lesions around the lower end of the DJ stent measuring 0.6–0.8 cm, 5 cases with diffuse erythema and hyperemia of the mucosa of the urinary bladder mainly at the anterior wall managed by randoms cold cup biobsy and 5 cases with elevated velvety red lesions of the mucosa.

. The associated allergic diseases were detected in 6 (27.27) patients (Table 1). Mean hemoglobin level was 12.4 + 2.4, ranged from 8 to 16.5 mg, only four patients had anemia. Mean White Blood Cells (WBCs) count was 7.4 + 2.3 thousands ranged from 3 to 17 thousands; two patients had peripheral eosinophilia (8.5% and 9.5%) but the rest of patients had normal eosinophil percent (up to 5%).

**Table 1:** Clinico-laboratory characteristics of the participants.

Characteristic	N (22)	%
Age (years)		
Mean ± SD	46.5 ± 17.5	
Range	22–78	
**Sex**		
Male	10	45.5
Female	12	54.5
**Symptoms**^a^		
Dysuria	17	77.3
Hematuria	14	63.6
Frequency	12	54.5
Urgency	2	9.1
Urine retention	2	9.1
**Clinical data**^a^		
Urinary tract infection	6	27.3
DJ stent	5	22.7
Chronic cystitis	5	22.7
Benign prostatic hyperplasia	3	13.6
Diabetes	2	9.1
Fatigue	2	9.1
Bilharzia	1	4.5
Hydronephrosis	1	4.5
Cancer	1	4.5
**History of allergy**		
No	16	72.7
Bronchial asthma	3	13.6
Skin allergy	2	9.1
Atopy	1	4.5
**Cystoscopic finding**		
Bladder mass	12	54.5
Erythematous area	5	22.75
Elevated mucosa	5	22.75
**Laboratory data**		
Haemoglobin (mean level)	12.4 ± 2.4 gm	
WBCs (mean count)	6.2 ± 2.6 (x1000)	
Eosinophil:		
Normal eosinophils (0–2%)	13	59.1
Normal eosinophils (2–5) %	7	31.8
High eosinophils (hypereosinophilia)	2	9.1

SD: standard deviation.

a One patients may have more than one symptom and more than one clinical data.

**Fig. 2:**
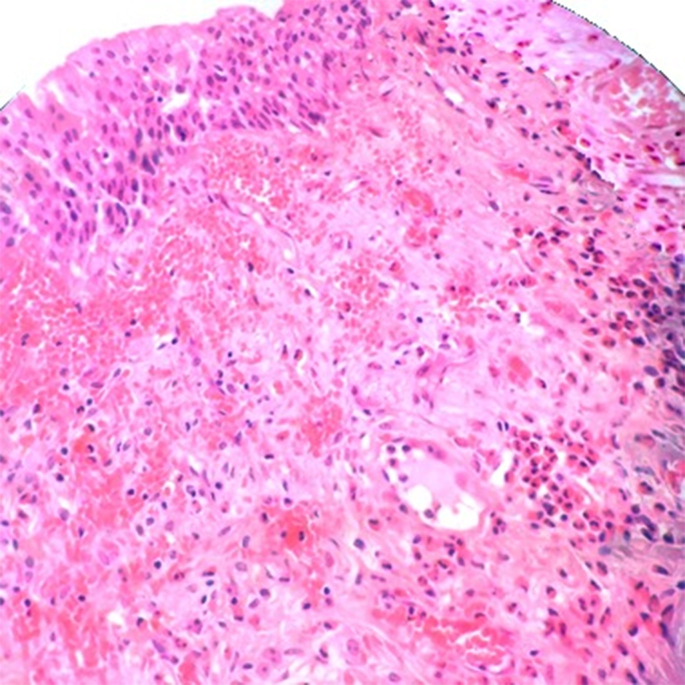
A histopathology picture of EC showing urothelial epithelium with underlying lamina propria exhibiting hemorrhage and inflammatory cells mainly eosinophils (H&E, 200x).

**Fig. 3:**
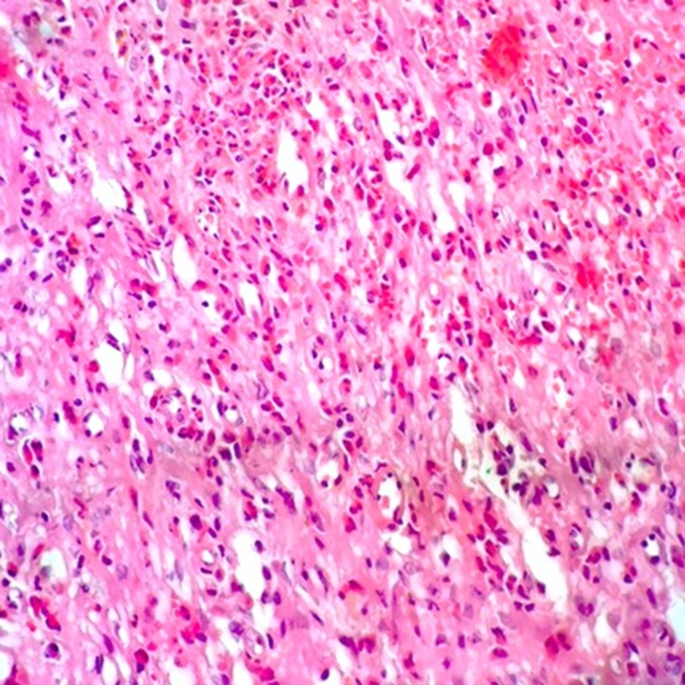
A histopathology picture of EC showing edema and inflammatory cells mainly eosinophils (>90%) (H&E, 400x).

Complete transurethral resection of bladder tumor (TURBT) was the predominant procedure performed for 13 (59%) patients and random cold cup biopsy procedure was done for the rest of cases. All cases were diagnosed as EC in the pathology laboratory depending on the predominance of eosinophilic infiltration (Fig. 2, Fig. 3), only 3 cases were diagnosed as acute phase and the rest of cases were reported as EC, without specifying acute or chronic phase. Medications were advised for all patients; The most commonly used medications were corticosteroids (prednisolone) for 72.7% of patients with tapering oral dose; 40,30,20,10 mg once daily tapered each 5 days for 1 month and maybe repeated up to 3 months, analgesics including non-steroidal anti-inflammatory drugs (NSAIDs) for 54.5% of patients, antihistamines including loratadine 10 mg once daily or chlorpheniramine once daily for 18% patients, and antibiotics for 27.3% of patients according to the culture and sensitivity test for UTI patients or the patients with dilatation were conducted. The majority of patients showed complete improvement (relief of all related symptoms) or partial improvement (Table 2). One recurrent case was recorded; the recurrence occurred following one year of the previous cold cup biopsy, she was advised for intravesical corticosteroids and oral antihistamine for 6 weeks for the recurrent lesion and showed a complete improvement with no other recurrence during the follow-up for 1 year.

**Table 2:** Outcomes of the medically treated patients.

Treatment	Number	outcome
**With Steroid** (tapering doses for 1 month at least)	16 (total)	
	12	Complete improvement
	4	Partial improvement
	1	Recurrence^a^
**Without steroids** (due to contraindication or patient's refusal)	6 (total)	
	2	Complete improvement
	4	Partial improvement
**Total**	22	P-value<0.05

a Recurrence occurs in a previously partially improved patient.

## 4 Discussion

Eosinophilic cystitis is a very rare inflammatory condition affecting patients of all ages, especial adults with a female predominance in some studies while others showing slight male predominance or equal sex incidence [10,11]. The results in this study established an average age of 46.5 + 17 years ranging from 22 to 78 years, with slight female predominance; 12 females vs 10 male patients. EC in children was reported in previous studies [12], we did not report any patients under age of 22 years old in this study, we detected two children with EC who were diagnosed during the study period but they are excluded from the study due to lack of completion of the follow-up period at our hospitals. EC has been proposed to be due to IgE-mediated activation of eosinophils following an antigenic stimulus which causes mast cell degranulation, and release of inflammatory mediators which damage the bladder wall. Allergy associated risk factors including atopic and environmental allergens can contribute to the disease [12].

The clinical manifestations of EC range from dysuria, frequency, urinary retention, and hematuria. The chronic condition characterized by pelvic pain/pressure which is perceived to come from the bladder, accompanied by at least one urinary symptom including urgency, frequency, and nocturia is called Bladder pain syndrome which has a range of pathophysiological mechanisms.

It is usually seen as a urinary bladder mass or thickness on sonography and on computed tomography (CT) urography making a differential diagnosis of tumors. The cystoscopic examination reveals papillary growth, tumor-like appearance, diffusely edematous mucosa, submucosal hemorrhage, or erythematous plaques. It is very difficult to distinguish EC from nonspecific cystitis and bladder tumor as the formation of mass lesion is confusing [10,13,14]. The most common presentation of patients in our study was dysuria in 77.3% of patients, followed by hematuria in 63.6% then frequency in half of the patients. In a literature review study, the most common recorded symptoms of the analyzed EC cases from 2003 to 2013 in the literature were frequency, dysuria, and urgency in 59% of cases, and urinary retention was reported in 11% of cases [15]. Manifestations of EC indistinguishably mimic those of neoplastic and other inflammatory bladder disorders that can precede or coexist with it [16].

The etiology of EC is unclear, but several hypotheses have been postulated in the associated conditions and the genesis of EC such as allergens, parasites, Bacillus-Calmette-Guerin (BCG), drugs, eosinophilic enteritis, recurrent urinary tract infection (UTI), transurethral resection of bladder tumor (TURBT), and bladder injuries [17]. In this study, we found 8 (36.3%) patients associated with UTI, and the associated allergic diseases were detected in 6 (27.3%) patients, in addition to 2 (9%) patients came with hypereosinophilia (Peripheral eosinophilia) without any given allergic diseases. Peripheral eosinophilia, defined as equal or more than 5% of total differential leukocyte count or 500 eosinophil cells per mm3 [18]. In previous studies [19,20], around 30% of EC cases were associated with peripheral eosinophilia which is much higher than our findings.

The second most frequent associated condition in this study was the urinary bladder injury in 6 (27.3%) patients (5 patients had a history of DJ stents and one patient had a history of previous urinary bladder surgery during the last two years).

Aboutaleb and Gawish [21], studied the histopathological changes due to DJ stenting, they found that acute eosinophilic reaction with mild edema was seen in DJ stent duration of fewer than 2 weeks, and the DJ stenting patients for 2–4 weeks, the biopsies examination showed acute lymphocytic EC with severe edema. This inflammatory reaction became more severe at 4–6 weeks, concluding that the use of DJ stent should be limited to highly indicated patients only.

It is imperative to obtain an adequate deep biopsy; otherwise, the diagnosis can be missed [10,22]. Histological examination of a transurethral biopsy from EC lesion shows edema, rarely plasma cells, lymphocytes, eosinophilic inflammatory infiltrate, with more prominent eosinophils within the lamina propria and muscularis or all the layers of the urinary bladder wall with few necrosis and more prominent eosinophilic cell infiltrate under the urothelial surface which is denuded in most areas. EC may be classified as either acute phase or chronic, according to fibrosis which increases in the chronic phase, however, the massive eosinophilic infiltrates are seen in the acute phase but mostly absent in areas of scarring in chronic phase. Giemsa stain can be helpful in the diagnosis of EC [10]. Due to the rarity of the disease, treatment is not standardized. The current treatment option for EC ranges from identification of an antigenic stimulus, supportive care, medical management with antibiotics, antihistamines, steroids, and surgical management including partial cystectomy and TURB resection [6] The role of steroids therapy as an anti-inflammatory drug has been emphasized in literature though the reported spontaneous resolution of symptoms. Cyclosporine and azathioprine as long as the other immunosuppressants have been used in case of no response to steroids [12]. Some patients were contraindicated for systemic steroid therapy and some refused steroids but the majority of patients were treated with steroids showing a significant improvement. However, recurrence still remains a risk that emphasizes the importance of follow-up of EC patients to detect the early recurrence of the disease. Limitations of this study lay in the limited sample size due to rarity of the disease and the other major limitation of the study is lack of full assessment of the associated allergic conditions. More studies on diagnostic approach and outcomes of the different therapeutic modalities are recommended.

## 5 Conclusion

Eosinophilic cystitis is an exceptional and rare pathology showing a misleading nonspecific presentation. Histopathology is the standard diagnostic tool exhibiting edema and inflammatory infiltrate with predominant eosinophils; however, careful differentiation between EC and other inflammatory diseases of the bladder is crucial. Medical treatment including corticosteroids is the first line with good prognosis, although recurrence remains a possibility which emphasizes the importance of patients’ follow-up.

## Ethical approval

Local Research Ethical Committee approval was obtained.

## Funding sources

This study did not receive any funding from public or private sectors.

## Registration of research studies

Clinicaltrials: NCT04914442.

## Provenance and peer review

Not commissioned, externally peer-reviewed.

## Declaration of competing interest

The authors declare no competing interests.
